# Recent Advances in the Biosynthesis of Polyhydroxyalkanoates from Lignocellulosic Feedstocks

**DOI:** 10.3390/life11080807

**Published:** 2021-08-10

**Authors:** Sevakumaran Vigneswari, Muhammad Shahrul Md Noor, Tan Suet May Amelia, Karthnee Balakrishnan, Azila Adnan, Kesaven Bhubalan, Al-Ashraf Abdullah Amirul, Seeram Ramakrishna

**Affiliations:** 1 Faculty of Science and Marine Environment, Universiti Malaysia Terengganu, Kuala Nerus 21030, Malaysia; vicky@umt.edu.my (S.V.); shahash632@gmail.com (M.S.M.N.); ameliasmtan@gmail.com (T.S.M.A.); kartthneebalakrishnan99@gmail.com (K.B.); azila.adnan@umt.edu.my (A.A.); 2 School of Biological Sciences, Universiti Sains Malaysia, Penang 11800, Malaysia; 3 Center for Nanofibers and Nanotechnology, Department of Mechanical Engineering, National University of Singapore, Singapore 117581, Singapore; seeram@nus.edu.sg

**Keywords:** polyhydroxyalkanoates, lignocellulosic, renewable carbon sources, waste, bacterial fermentation, biopolymer

## Abstract

Polyhydroxyalkanoates (PHA) are biodegradable polymers that are considered able to replace synthetic plastic because their biochemical characteristics are in some cases the same as other biodegradable polymers. However, due to the disadvantages of costly and non-renewable carbon sources, the production of PHA has been lower in the industrial sector against conventional plastics. At the same time, first-generation sugar-based cultivated feedstocks as substrates for PHA production threatens food security and considerably require other resources such as land and energy. Therefore, attempts have been made in pursuit of suitable sustainable and affordable sources of carbon to reduce production costs. Thus, in this review, we highlight utilising waste lignocellulosic feedstocks (LF) as a renewable and inexpensive carbon source to produce PHA. These waste feedstocks, second-generation plant lignocellulosic biomass, such as maize stoves, dedicated energy crops, rice straws, wood chips, are commonly available renewable biomass sources with a steady supply of about 150 billion tonnes per year of global yield. The generation of PHA from lignocellulose is still in its infancy, hence more screening of lignocellulosic materials and improvements in downstream processing and substrate pre-treatment are needed in the future to further advance the biopolymer sector.

## 1. Introduction

The highly flexible petroleum-based plastics are an inevitable commodity in our everyday life [1]. Other materials such as (glass, metal, and wood) have been long replaced by petroleum-based plastics, across various industrial sectors, for different applications. This happened because plastics have chemical and physical properties that provide them with strong durability, flexibility, cost-effectiveness, and a wide variety of applications and versatility. However, when they are disposed of in the environment, their characteristics turn plastics into contaminants. In the field, plastics degrade at a very slow rate, with a half-life of up to 500 years, whereas untreated bio-based plastics usually degrade within a range from two weeks to a year while also depending on the environment, since biodegradation would be extremely slow in sterile conditions [1]. In addition, Urtuvia et al. also reported that the weight of plastic waste is around 8%, and the amount of total solid municipal waste is 25% [1]. The low biodegradation, insufficient waste management, and lack of environmental awareness have resulted in the accumulation of plastics in the environment. Plastic waste is known to be a major environmental pollutant causing problems with waste management. It takes decades for traditional plastics to decompose and develop toxins during the process of degradation [2]. In 2013, plastic production contributes 299 million tons. Compared to 2012, 2013 is estimated to be 3.9% higher, causing significant environmental problems [2]. Output has been growing continuously over the years. For instance, the decomposition in nature of traditional plastic (polyethylene, polypropylene) takes around 20–100 years, creating numerous problems such as environmental contamination, water pollution, and air pollution, which are aggravated by the recycling and incineration of plastics that could release poisonous gases [2]. Furthermore, the existence of plastic particles in oceans affects marine life at large. However, they come with serious problems such as their poor biodegradation and accumulation in the environment. Thus, plastic processing, utilising renewable feedstock and the production of new bio-sourced plastics, has been advocated in the past few decades to reduce the production of conventional plastics, which is also the root and main factor of fossil-based plastic recycling. Subsequently, the production of bio-based plastics known as biodegradable polymers is of growing interest because it possesses similar physiochemical properties to synthetic plastics [2].

Polyhydroxyalkanoate (PHA) is a type of biodegradable plastic that can be acquired as a source of carbon and energy by many kinds of bacterial genera [3]. With additional benefits, PHA has similar properties to conventional plastics, which are biodegradable, sustainable, and biocompatible [4]. The life cycle of bioplastics typically begins with the fermentation of sugars, lipids, or renewable carbon materials to produce PHA, which is then manufactured based on its applications. With an average of 100% biodegradation in 90 days, the end products are naturally biodegradable into carbon dioxide and water. Discarded PHA products can be recycled or composted for use by PHA carbon feedstocks, thus practising the cradle-to-cradle concept [5,6]. Therefore, to solve environmental issues created by waste disposal, biodegradable plastics are a possible alternative [7]. PHA has many unique characteristics, such as strong UV resistance, antioxidant capabilities, gas barrier properties, optical conductivity, non-toxic features, as well as thermoelectric properties, which are commonly applied in medical, tissue engineering, electrical components, and packaging [8]. At present, the PHA industrial development is primarily carried out by pure bacteria or recombinant bacteria, which have the benefits of a high rate and large yield of synthesis [3]. That being said, the production of pure genera of bacteria that require controlled circumstances during the operation and the utilisation of special feedstock, has made the production cost of PHA 4–9 times more than conventional plastics, resulting in limitations of the industrial application of PHA [9]. The cost of sugars used for production is the main limiting factor for its commercial success and it has been estimated that 3 tonnes of glucose are needed to produce a tonne of PHA.

**Figure 1:**
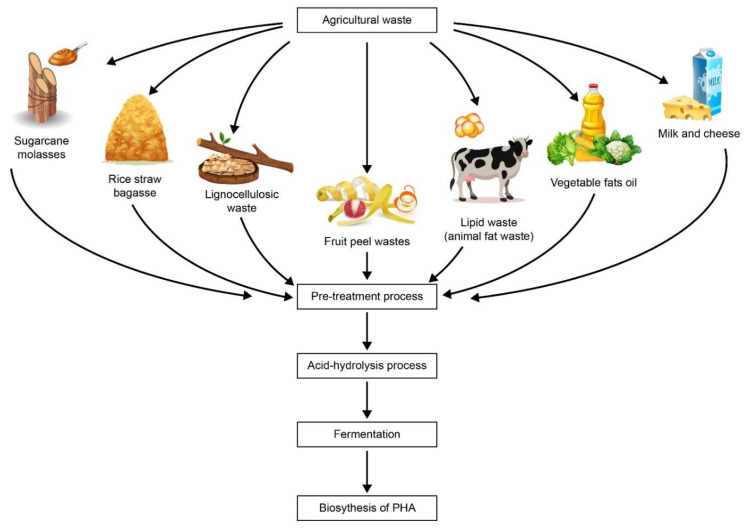
Various food processing and agricultural industries are a good source of renewable feedstock for the production of microbial bioplastics such as polyhydroxyalkanoate (PHA).

Moreover, the world is heading toward a circular and bio-based economy. This necessitates a strong emphasis on the efficient and long-term use of bioresources. These low-value waste materials include (i) sugar-rich derivatives (sugar-producing sap/juice plants and sugar-rich by-products from sugar-producing industries); (ii) sufficient feedstocks of triacylglycerols and fatty acids (edible, non-edible waste oils, crude glycerol, and industrial wastewater), and (iii) cellulosic raw materials (lignocellulosic biomass hydrolysates, corn steep liquor, brans, and straws). These waste feedstocks, second-generation plant lignocellulosic biomass, such as maize stoves, dedicated energy crops, rice straws, wood chips, are commonly available renewable biomass sources with a steady supply of about 150 billion tonnes per year of global yield [10]. Additionally, the use of first-generation sugar-based cultivated feedstocks for PHA production, such as food crops, whey, molasses, sugar cane, corn sugar, palm, and vegetable oils. The use of the first-generation sugar-based cultivated feedstocks for PHA production threatens food security due to global population demands for maximum utilisation of feedstocks. Besides, the use of these feedstocks depends on enormous quantities of land, water, chemicals, and energy for development, harvesting, transport, and processing. In addition, the use of these edible feedstocks in the polymer industry decreases their food supply chain availability and increases food prices which further hinders their widespread commercialization. Waste is generally defined as any unwanted material, such as leftovers that are not fully utilised from food consumption, and the production of new products [10]. Human activity generates massive quantities of waste in the environment. According to Brojanigo et al., waste comes from various sources, including agricultural waste (crops, orchards, lignin, cellulose, and hemicellulose), industrial waste (fabrication, manufacturing, industrial processes and building sites), municipal waste (household waste, landscaping, and wastewater treatment plants), institutional waste (government centres, schools, and hospitals), and commercial waste (restaurants, stores, and markets) [11]. Sathya et al. have described that most of these agricultural wastes are obtained from major commodity crops in large quantities [12]. Moreover, food processing and agricultural industries throughout the years can be good renewable feedstock for bioplastic production (Figure 1). Exploitation and maximum usage of agricultural residues reduce the substrate cost as well as managing the costs of waste disposal. Brojanigo et al. stated that there are many agricultural wastes that can have strong potential for substrate replacement, such as fruit peels, pawpaw fruit peels, oil palm, maize cobs, maize chaff, bagasse hydrolysate, sugarcane bagasse, soya flour, rice bran, waste glycerol, waste plant oil, and animal fats [11]. Forests are a key source of lignocellulosic biomass with promise in a variety of industries because of their high availability, long-term utilisation, and good management. Lignocellulosic waste such as sugar cane bagasse, cereal straw, and others are also significant sources of feedstocks in areas where forestry supplies or leftovers are scarce [13].

Non-food agroforestry resources such as lignocellulosic feedstock (LF) (e.g., wood, and agricultural residues) can also be used to make PHA. Some recent projects have seemed to generate second-generation biodegradable plastics from different agricultural residues [14]. The separation of lignocellulosic from forestry biomass into the primary components, cellulose, hemicelluloses, and lignin, is required. Compared to bioplastics made from corn, starch, or sugars, although bioplastic biosynthesis from LF is more demanding, the latter poses a significant opportunity to minimise the usage of petroleum-based polymers. This is significant since one of the most critical factors for the bio-based industry’s long-term success is the creation of advanced bio-based materials [15].

Consequently, PHA has been commercially successful in many fields of application such as in the biomedical, agricultural, and industrial sectors [8,15]. The use of adequate raw material as a carbon source is essential to produce PHA on a large scale. However, much research has been conducted to investigate the effectiveness of renewable carbon sources, such as LF, that is abundant around the world but has been poorly studied due to its challenges. It is crucial because if the production of these bioplastics using LF as raw material can be proven, it can replace expensive carbon sources and increase the yield of this potential biopolymer as well as lower the cost of overall production at the same time. The commonly used bacterial strain that can utilise this feedstock has to be found in an investigation. Thus, this review aims to highlight most of the bacterial strains that produce high PHA yield from LF throughout the last decade.

The utilisation of LF to replace non-renewable carbon sources in PHA production industries minimises the cost of PHA production in the future to sustain the current and future production of biomaterial, biofuel, and biomolecules. This review will highlight recent studies regarding PHA production from renewable feedstocks, the recent and latest improvements in PHA industries, and address the research gaps for more future improvements in PHA production using LF. The novel bacterial strains that use LF as PHA as a substrate will provide a substantial and vital contribution towards the cost incurred in the PHA production thus contributing to scientific research and environmental sustainability.

## 2. Current Situation of Global Plastic

Plastics are used in many aspects of our daily lives nowadays. In most cases, “plastics” are polymers derived from petrochemistry. Plastics are the most rapidly emerging group of materials applied for the manufacturing of customised items because of their properties such as high resistance and low density. Nevertheless, due to a lack of petrochemical resources and plastics’ resistance to biodegradation, traditional petrochemical plastics are causing an increase in global concern. Geyer et al. estimated 8–9 × 10^9^ tonnes of plastics were produced globally in recent decades [16]. Due to plastics’ high resistance to biodegradation, other disposal techniques are typically used, such as simple landfilling or simply dumping in the surroundings; this fate befell about 79% of plastics produced, resulting in growing piles of plastic distributed globally [16,17]. The most unsolved problem is critical marine pollution, where about 2 × 10^6^ tonnes of plastic waste entered the undersea ecosystem through coastal sewers and rivers, endangering the food chain via microplastics [17]. Not only renewable and biocompatible but also biodegradable and compostable PHA is a plastic-like material that is different from fossil-based plastics, therefore, its use has been considered to be environmentally friendly and sustainable [8,15]. However, public awareness regarding the different types of plastics and their properties is likely crucial to driving material substitution, where possible, from fossil-based plastics to eco-friendly bio-based plastics such as PHA. Optionally, approximately 12% of the plastics manufactured today have been combusted for energy production, resulting in the creation and release of greenhouse gaseous emissions [16]. Recycling using petrochemical plastics, on the other hand, is a widely accepted and innovative technology. The average production costs of bioplastics far outweigh the average recycling costs of petrochemical plastics by an estimated USD 5000 per tonne [18,19]. However, this alternative is limited due to the requirement for waste plastic to be of a specified purity in terms of the type of pollution and plastic as well as a quality loss with each cycle and exhaustion of material [20].

Considering current global plastic manufacturing of nearly 4 × 10^8^ tonnes per year, with an enormous uptrend observed, characterised by increasing industrialisation, it is clear that massive amounts of plastics waste (now exceeding 15 × 10^7^ tonnes per year) require immediate disposal [20]. Table 1 shows the uprising amount of plastic produced globally.

Due to plastics’ high resistance to biodegradation, other disposal techniques are typically used, such as simple landfilling or simply dumping in the surroundings; this fate befell about 79% of all plastics ever produced, resulting in growing piles of plastic, and distributed all over the world. The most unsolved problem, critical marine pollution, where about 2 × 10^6^ tonnes of plastic waste entering the undersea ecosystem through coastal sewers and rivers endangered the food chain via microplastics [17]. Optionally, approximately 12% of plastics manufactured today have been combusted for energy production, resulting in the creation and release of greenhouse gaseous emissions [16]. On the other hand, the conversion of plastic waste into bioplastic is also faced with difficulties such as yield productivity [22]. Recycling using petrochemical plastics, on the other hand, is a widely accepted and innovative technology. However, this alternative is limited due to the requirement for waste plastic to be of a specified purity in terms of types of pollutions and plastics as well as a quality loss with each cycle and the exhaustion of material [20].

### Bioplastics

Bioplastics are naturally occurring biodegradable materials derived from renewable biomass resources, which can serve as alternatives to petroleum-based plastics. Biopolymers are of biological origin as we discover various types of natural biopolymers including DNA molecules and proteins as natural rubber, starch in grains, and sometimes even cellulose in plants. According to Yousuf, bioplastics can be synthesised by the chemical polymerization of monomers or directly through fermentation by plants and microorganisms [23]. For instance, natural rubber is a biopolymer that can be extracted from the *Hevea brasiliensis* tree as a latex. Biodegradable plastics can be categorised into bio-based plastics or biodegradable plastics, depending on their exact composition [21]. Classically, Getachew and Woldesenbet [24] stated that biodegradable plastics are typically made up of fossil products, whereas bioplastics are metabolised from sustainable resources of biomass, such as food waste, sawdust, corn starch, and agricultural oils and fats. Bio-based can usually be obtained from agricultural waste using microorganisms. Maraveas concluded that environmentally friendly or biodegradable plastics derived from renewable carbon provides benefit as they allow sustainable use of agricultural waste, improve soil fertility at a good rate of biodegradability, and reduces environmental burden imposed on consumers [25].

**Figure 2:**
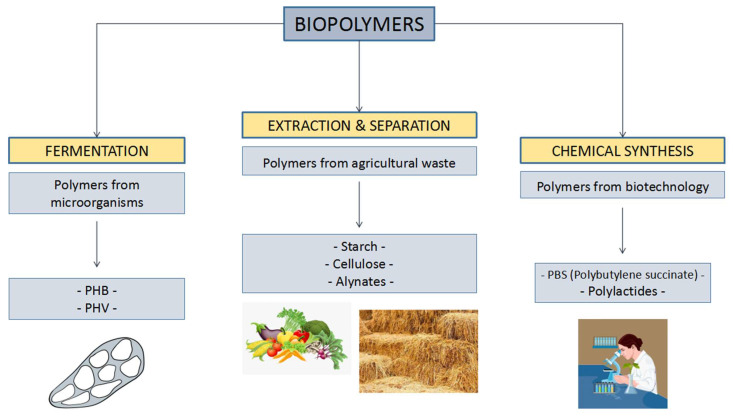
Various methods employed in the production of biopolymers.

According to Alves et al., photo biodegradable plastics, completely biodegradable plastics, and semi-biodegradable plastics are the three main types of biodegradable plastics [26]. Photodegradable plastic acts as additives with the light-sensitive polymer chain groups. According to Koller, exposure to ultraviolet radiation for a long period causes the degradation of their polymeric structure to occur, followed by complete biodegradation [27]. Koller stated that these plastics remain non-degraded when landfills are exposed to less sunlight [27]. Semi-biodegradable starch-linked plastic contains more additional short polymers that are non-biodegradable. However, the fragments of polyethylene, as a non-starch portion of the polymer, prevent the plastics to be degraded by the bacteria [28]. Figure 2 shows the classification of biopolymers based on production [13].

Based on Figure 2, biopolymers can be divided into different classes based on their production; for example, polymers that are produced by bacteria undergo a fermentation process to produce polyhydroxybutyrate (PHB) and polyhydroxyvalerate. Secondly, polymers that are obtained by the extraction and separation of agricultural waste products produce starch, cellulose, and alynates [13]. Additionally, polylactides and polybutylene succinate are polymers from biotechnology obtained by the conventional synthesis process [28].

## 3. Polyhydroxyalkanoate (PHA)

PHA is a biodegradable, natural polyester produced as intracellular granules by bacteria as storage for energy under insufficient nutrients [29]. External factors such as oxygen, nutrients, as well as internal limits are intended for these conditions. These polymers have a chemical structure that is almost the same as petroleum-based plastics, hence having similar physico-chemical properties. PHA is acknowledged due to its high biodegradability, biocompatibility, and sustainability [30]. According to Thakur et al. and Tan et al., there are over 150 monomeric building blocks [31,32]. It was found to have a future as PHA synthases substrates and polymerisation. It was discovered that it might be used as a substrate for PHA synthases for polymerisation. However, PHA has only been utilised in research environments, and only a few have been applied in the industrial field. Surprisingly, only R-configured PHA is found in nature, and most building blocks are chiral because PHA synthases are stereospecific. The first time these polymers were found was in 1888. Furthermore, at that time, it was not possible to properly describe their composition and biological function. As early as 1926, a French scientist obtained poly-3-hydroxybutyric acid, P(3HB), from *Bacillus megaterium*, and Maurice Lemoigne discovered PHB as an internal bacteria granule for *Bacillus megaterium*, which was the first PHA [33,34]. Keshavarz and Roy stated that PHB is the most well-studied and proven among all the PHA types and used as a reservoir product in microbes attributable to up to 80% of the dry biomass of microbial [35].

### 3.1. Structure and Classification of PHA

**Figure 3:**
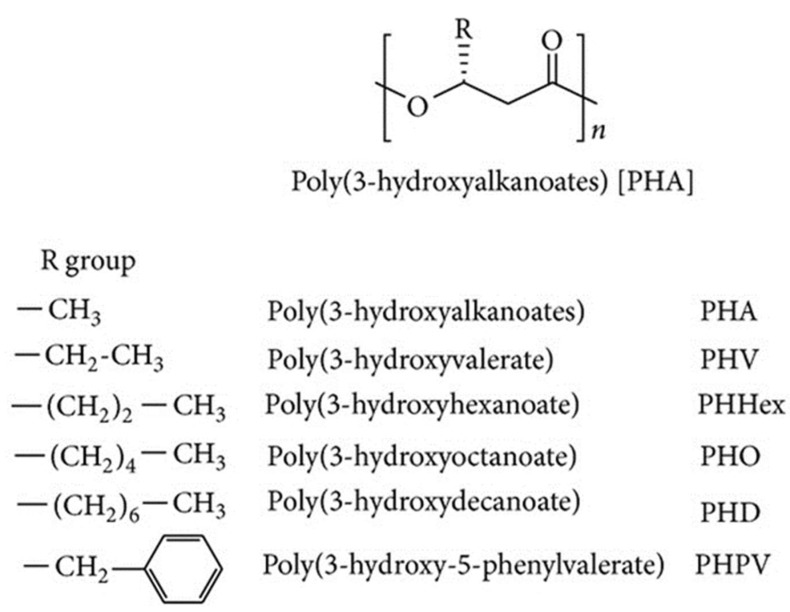
General structure of polyhydroxyalkanoate (PHA) and examples of their structural derivatives [38].

There is a side chain (R) in any PHA monomer, mostly a saturated alkyl monomer. However, it may also consist of branched alkyl, unsaturated alkyl, and replaced alkyl groups [34,36]. At present, PHA has about 150 different components of PHA monomers that are recognised based on the length of the carbon chain and their structure of attachment, such as branched, straight, aromatic, unsaturated and saturated [37]. The general structure of PHA is shown in Figure 3.

PHA usually has a similar formula in which different groups of R hydroxyalkanoic acid are attached. PHA is classified into three groups (Table 2), the arrangement and number of carbon atoms in the chain, along with their branching [39].

However, in nature, LCL PHA is not widely known [44]. There is disparity between SCL PHA and MCL PHA primarily due to the substrate specificity of some PHA synthases that only polymerise certain 3-hydroxyalkanoate (3HA) of a limited range of carbon numbers. For instance, *A. eutrophus* can polymerise 3HA of 3 to 5 carbon atoms while *Pseudomonas aleovorans* PHA synthase only can recognise 3HA of 6 to 14 carbon atoms [41]. Anjum et al. also stated that the hybrid polymers containing both group monomeric units, such as polymeric units (3-hydroxybutyrate-*co*-3-hydroxyhexanoate), also exist [41]. It is due to both monomers being in the R-configuration, the stereospecificity of the biosynthetic enzymes. Additionally, homopolymer and heteropolymer are also well-recognised categorisations of PHA.

### 3.2. Properties and Application of PHA

**Figure 4:**
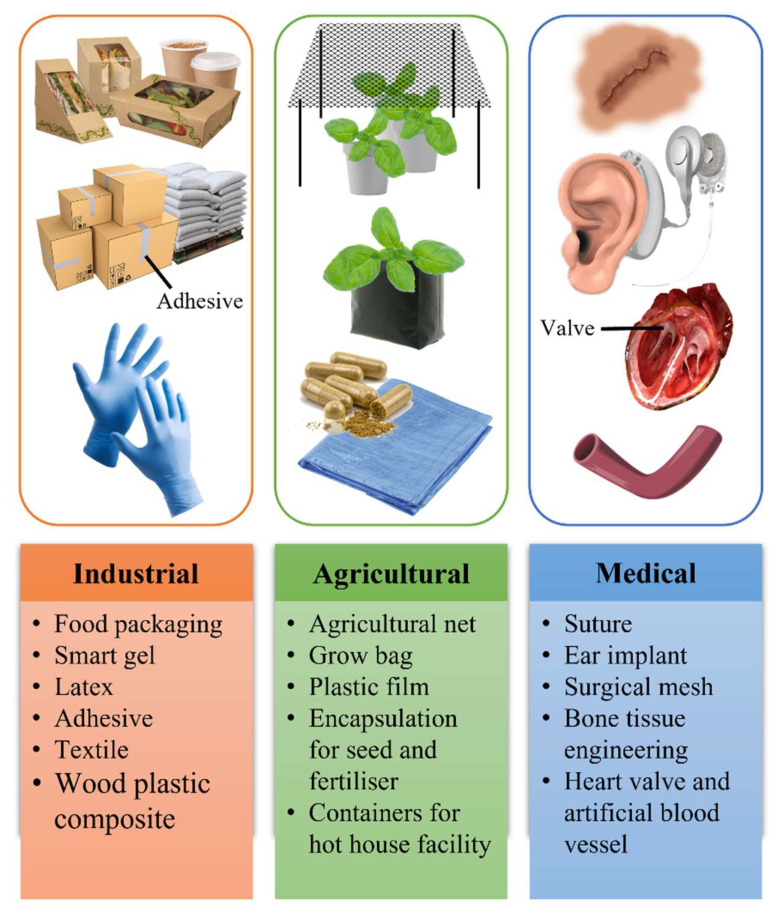
Commercial applications of polyhydroxyalkanoate (PHA) in various sectors regarding its biodegradability and biocompatibility.

Due to the variations in the structural and chemical compositions of PHA, the physico-chemical characteristics of PHA are different from one another. Bugnicourt et al. reported that PHA is water-insoluble, which helps to prevent hydrolysis [45]. In the absence of oxygen in strike, sedimentary soil and its sinking characteristics in water enhanced biodegradation [45]. In addition, they are very biodegradable, inherently piezoelectric, and biocompatible [45]. PHA is more soluble in chlorinated solvents while insoluble in non-chlorinated solvents. PHA has a transition glass temperature and melting temperature range of −50 °C to 4 °C and 40–180 °C, respectively reported by Czerniecka-Kubicka and co-workers [46]. PHA also showed physico-chemical properties including breaking strength, thermal degradation, vapor content, and elasticity modulus, which are highly dependent on the biopolymer’s composition [37,45]. The basic characteristics of PHA make this polymer a vital biochemical material in multiple areas with different applications (Figure 4).

### 3.3. PHA-Producing Bacteria

**Figure 5:**
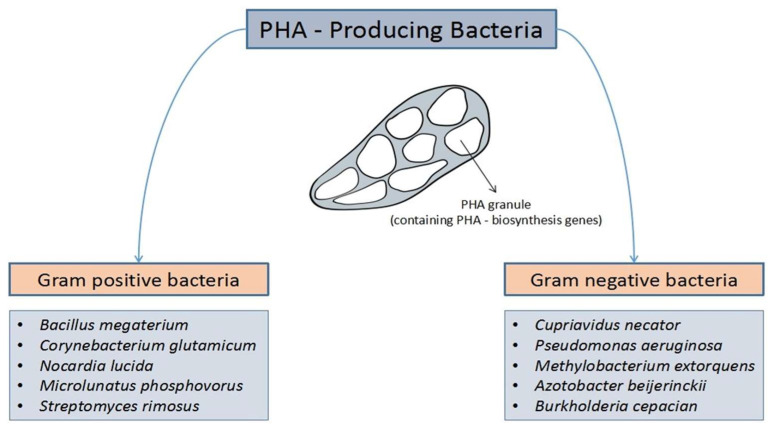
Common examples of the various Gram-positive and Gram-negative bacteria producing polyhydroxyalkanoate (PHA).

The bacterial production culture for PHA is more comparable to living organism production, especially plants, from an economic aspect its greater capacity for accumulation [37]. Vaneechoutte et al. reported that bacterial species that were commonly studied for the biosynthesis of PHA were *A. eutropha*, *R. eutropha*, and *Cupriavidus necator* (Figure 5) [47]. Other possible bacterial strains, such as *Pseudomonas* sp., *Bacillus* sp. *Rhodopseudomonas palustris*, *Burkholderia sacchari*, *Halomonas boliviensis*, and *Aeromonas hydrophilia* have recently been investigated for PHA synthesis based on yield [37].

PHA-producing bacteria can be classified based on nutrient stress, growth pattern, and requirement of nutrients [37]. Under this classification, the bacteria have been classified into two groups. The first group is the prime group where the bacteria that belong to this group store PHA, they require restricted nutrients such as oxygen, magnesium, phosphorus, and nitrogen and are unable to biosynthesise PHA during their growth time [48]. Examples of bacterial species that belong to this group are *Pseudomonas putida*, *Pseudomonas oleovorans* and *Ralstonia eutropha*. 

Otherwise, the production of PHA by the second group is not influenced by the supply of nutrients and is also able to continue to maintain PHA during its growth stage [49]. Nitshke et al. reported that mutant strains of *Azotobacter vinelandii*, recombinant *Escherichia coli* and *Alcaligenes latus* are examples of the bacteria species that belong to this second group [50].

## 4. Carbon Sources or Feedstocks for PHA Production

The source of carbon plays a vital role in the development of PHB costs and adds to 50% of the overall cost of manufacturing [2]. Processes of production using low-cost raw materials such as residues from the agro-industry for the manufacturing of PHA. A wide variety of substrates including industrial substrates by-products, oils and fats, lignocellulose material, household and agricultural waste, and sugar have been used [41]. The substrates that are commonly utilised to produce PHA are from the agro-industrial wastes such as soy-bran and hydrolysates that are reported by Anjum and co-workers [41]. The production of PHA using various strains of bacteria and substrates are listed in Table 3.

### 4.1. Waste Feedstocks

PHA has been commonly generated from municipal wastewater, solid waste, and cheese whey [52,66], waste cooking oil [57,67,68,69], cane molasses [70], spent coffee grounds [71], phenol [72], food waste [73], sweetwater, a by-product of processing sugar cane [74], non-food crops such as ryegrass [75], and agro-industrial waste [59]. Although various wastes have been reported to be recoverable to PHA, agricultural crop residues are the most available and investigated carbon substrate that helps in the growth of bacteria culture and PHA production [73]. The main focus that Pakalapati et al. showed was on transforming rural, food-derived waste and industrial into PHA [15]. As proven in the study, there are three main pathways for synthesising PHA (pathway I, acetyl-CoA to 3-hydroxybutyryl-CoA, pathway II, fatty acid degradation and pathway III, fatty acid biosynthesis) [76]. Two types of waste have been identified based on these pathways [77]: sugar waste and fatty waste. Rodriguez-Perez et al. stated that waste-containing sugar and/or fatty acids may be the best feedstocks for PHA production [78]. Based on Chee et al., the PHA yield produced using fatty acid substrates and sugar substrate as feedstocks were around 0.6–0.8 g/g and 0.3–0.4 g/g, respectively [79].

### 4.2. Lignocellulosic Feedstocks (LF)

With an estimated global quantity of approximately 200 billion tonnes, LF has been widely known as the most abundant organic materials on earth [80] with compositions of cellulose (40–50%), hemicellulose (20–50%), lignin (20–30%), phenolic component, and extractives [81,82]. Similar to waste feedstock, the pretreatment process and hydrolysis are necessary for obtaining results such as increased availability of carbon sources, diluted organic matter concentration, pH regulation, temperature control, sterilisation of waste materials, elimination of foreign solids, and minimisation of possible effects of inhibitors, such as furfural, on production strains for PHA [78]. The pretreatment processes that involve the physico-chemical, biological reactions, and hydrolysis of various LF have achieved high amounts of sugar yields to produce biofuels over the past decade under existing biorefineries as stated by Zhang and co-workers [82], while hydrolysis creates a stream of carbon containing primary sugars, microbial inhibitors, organic acids, and derivatives of lignin [83]. Tomizawa et al. focused on biopolyester production from derivatives of lignin, while Sandhya et al. reported *Ralstonia eutropha* as a strain to produce PHA and PHB from lignocellulosic biomass [84,85].

### 4.3. LF for PHA Production

The lignocellulosic biomass can be categorised based on its source, content, and structure [86]. Biomass can be generally classed as woody materials, perennial bioenergy crops, agricultural residues, and municipal solid wastes, though classification varies based on circumstances (Table 4).

Bertrand et al. completed the first PHA production research employing lignocellulosic hydrolysate as a carbon source in 1990 [88]. The scientists found that the pentose in the hydrolysate from the hemicellulose fraction of poplar wood could be used by *Hydrogenophaga pseudoflava* ATCC 33668 also known as *Pseudomonas pseudoflava*. However, when compared to other resources such as sugar and oils, the PHA yield from LF remained low [89]. 

The monomeric sugars in hemicellulose vary between hard and softwood, but the structure of cellulose and lignin appears to be species-dependent. As an example, hard-wood lignin consists mainly of S and G units, and softwood lignin mostly consists of G units. Herbaceous lignin, on the other hand, includes H, S, and G units [90]. Wood residues contain little water and are often a clean, homogenous material free of contaminants. Despite extensive study in the field of pretreatments, converting woody biomass into cost-competitive products such as ethanol remains an economically complex process. Pan et al. achieved the greatest results so far by producing a maple hemicellulosic hydrolysate (containing 72 g/L xylose) and using it as a feedstock for PHA synthesis [91]. A biomass concentration of 17 g/L containing 51% PHA was achieved in a fed-batch fermentation utilising *Burkholderia cepacia*. According to the authors, a productivity of 0.09 g/L/h is poor, but it might be enhanced by optimising the feeding method.

Talebian-Kiakalaieh et al. described that the *B. cepacia* ATCC 17759 bacteria strain tested on sugar maple hydrolysate produced 51% of 3-hydroxybutyrate (3HB) [92]. The utilisation and conversion process of lignocellulosic and cellulosic waste into bioproducts enhances the PHA productivity [81]. However, due to the strong cohesion of LF components, the procedure of separating the compounds such as lignin, cellulose, and hemicellulose could be tedious [93]. According to Preethi et al., when *Ralstonia eutropha* was explored using Jambul seed, *Syzygium cumini*, the PHA production was 41.77% of cell dry weight (CDW) with (0.044 g/L) [28]. For effective production of PHA production, cellulose hydrolysates have been utilised as carbon sources. Based on a study conducted by Nduko et al., *E. coli* LS5218 has been used to obtain 59% of CDW with (3.3 g/L) of 3HB from cellulose hydrolysates [94]. Moreover, sunflower stalk hydrolysate tested with *E. coli* HMS174 (DE3 produces 89% of CDW with (16.2 g/L) of poly(3-hydroxypropionate-*co*-3-hydroxybutyrate) P(3HP-*co*-3HB) production and *Eucalyptus* hydrolysate cultured with *E. coli* produced 62% of CDW with (5.4 g/L) of poly(3-hydroxybutyrate-*co*-lactate) P(3HB-*co*-LA) [95]. For example, *Cupriavidus necator* H16 were screened for PHA production from bagasse hydrolysate and wheat bran hydrolysate producing PHA contents of 54% and 3HB of 66%, respectively [13,96]. Favaro et al. showed that sugarcane bagasse hydrolysate tested with *B. cepacia* IPT 048 produces 53% of CDW with (2.3 g/L) of 3HB [80]. Aremu et al. showed that cassava starch hydrolysate tested with *Pseudomonas aeruginosa* NCIB 950 produces 57.7% of PHA [97]. The *Burkholderia sacchari* DSM 17165 strain was originally used for the direct conversion of wheat straw into P(3HB-*co*-4HB) with a concentration of 37% of CDW with (0.29 g/L) [61]. Obruca et al. stated that two *Burkholderia* sp., which is *B. cepacia* IPT 048 and *B. sacchari* IPT 101, strains showed interesting performances in xylose and glucose producing 3HB contents of 57% and 58% of CDW, respectively [98].

Cesário et al. obtained the best results on concentrated wheat straw hydrolysate in the scholarly literature [99]. They were able to achieve a biomass concentration of 146 g/L with 72% P(3HB) at a productivity of 1.6 g/L using *Burkholderia sacchari*. *Cupriavidus necator* hydrolyses cellulose from agricultural straw leftovers and converts it to PHA. Rice straw, corn stover, or distiller grains are used as carbon sources in the second invention [100]. However, the relevant enzymatic and conversion studies should also consider the possible presence of inhibitors that could influence the enzymatic rate [80]. Overall, the use of LF in PHA production is still ongoing research (Table 5) [101,102,103].

## 5. Conversion Processes of LF for PHA Production 

**Figure 6:**
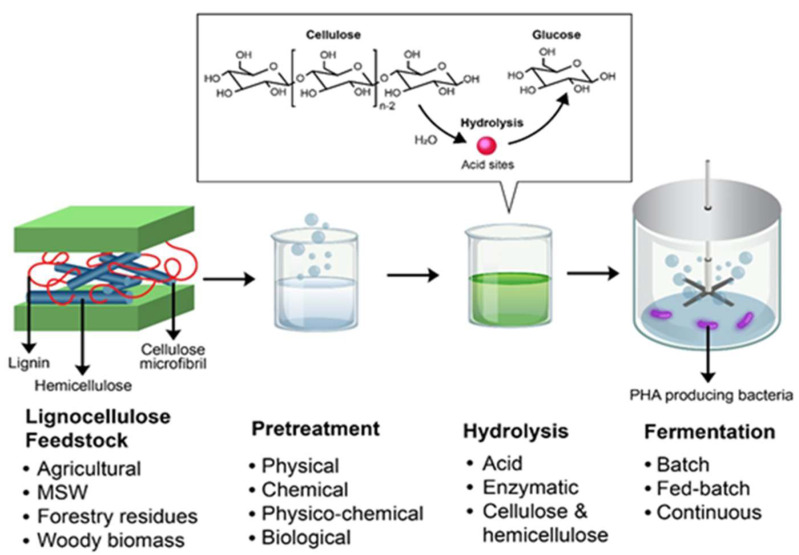
Biotransformation of lignocellulosic feedstocks (LF) into polyhydroxyalkanoate (PHA) includes several steps such as pretreatment and hydrolysis before it can be used as a carbon source in the production of PHA.

LF are mainly polysaccharides that can be converted into fermentable sugars or chemically altered into valuable by-products. LF is abundant, cheap, and it does not compete with the human food chain, hence a promising substrate for PHA production. However, conversion of lignocellulose materials to yield fermentable sugars as substrates for PHA production pose a challenge due to many factors, such as hydrolysis and pretreatment of the biomass. The conversion of LF into fermentable sugars for PHA production is depicted in Figure 6. Despite the fact that several research studies have been conducted on the production of PHA from LF, due to its antioxidant and antimicrobial structure, its effective utilisation remains a challenge, which makes the conversion of cellulose and hemicellulose into monomer sugars difficult [122].

### 5.1. Pretreatment

Several factors affect LF conversion, including lignin and hemicellulose safety, accessible surface area, hemicellulose acetylation, as well as the degree of cellulose polymerization and cellulose crystallinity [123]. As a result, pretreatment is an essential procedure since it speeds up the breakdown of the lignin-carbohydrate complex into sugar [124]. This process is important in triggering major changes in size, chemical composition, and chemical assembly, leading to a more effective hydrolysis process and higher amounts of yield [104]. On the other hand, the lignin and xylooligomers produced during this process can prevent the enzymatic hydrolysis via irreversibly binds to enzymes [125]. As a result, the pretreatment process must be non-specific to the feedstock, requiring less chemical, water, and electricity. To maximise the enzymatic rate in hydrolysis and the yield of sugars, the feedstocks must be highly reactive at both micro and macro-accessible sites. However, not only are the chemicals used (e.g., concentrated acid) harmful to the environment, but the biomass refining process for higher yield could further contribute to the operation costs [80]. Thus, several pretreatments have been studied throughout the past and recent years (Table 6) [126].

According to Wang et al., pretreatment of newspaper and office waste resulted in an enormous increase in glucose output [127]. The glucose yield of office paper processed with diluted acid increased from 69% to 91%. When compared to untreated paper, ammonia fibre expansion (AFEX) preparation resulted in a 13% rise in glucose concentration and a 50% rise when applying oxidative lime [128].

Ionic liquid (IL) has recently attracted a lot of interest in the lignocellulose biorefinery idea because it can fractionate biomass into lignin-rich materials, hemicellulose, and carbohydrate-rich materials [129]. Furthermore, IL is a replaceable material with negligible activity loss [129,130]. 

According to Wu et al., IL pretreatment of poplar wood with 1-ethyl-3-methylimidazolium acetate enhanced enzymatic digestibility, decreased cellulose crystallinity, and resulted in full cellulose hydrolysis at extremely low enzyme loadings [131]. While microwave pretreatment of pinewood with dimethyl sulfoxide resulted in a considerable increase in enzymatic hydrolysis of 85.4% [132]. The advantages and disadvantages of other pretreatments are listed in Table 7.

### 5.2. Hydrolysis

In the LF, cellulose and hemicellulose can be hydrolysed through enzymatic or chemical hydrolysis to produce sugars including glucose and xylose, which could be used to make value-added chemicals [137]. The traditional and commonly used methods in practice are acidic and enzymatic hydrolysis of LF.

Acid hydrolysis is a common and well-known used method for LF, as it can permeate lignin and break down both hemicellulose and cellulose to yield simple sugars without the need for any pretreatment [128]. According to Lenihan et al., any diluted (2–5%) or concentrated (10–30%) of nitric acid, sulfuric acid, hydrochloric acid, formic acid, and phosphoric acid and can be used to induce this hydrolysis [138]. However, both of these (concentrated and diluted) acid hydrolysis processes come with limitations as concentrated acid needs a moderate temperature range to produce a high yield of glucose (90%) and contribute to oxidation and other damages [138].

Hemicellulose and cellulose are degraded via hydrolysis during dilute acid pretreatment and soluble sugars are gained. Generally, the hydrolysis of hemicellulose produces sugars such as glucose, mannose, and galactose. Under acidic conditions, cellulose can be degraded to monosaccharides such as glucose. Subsequently, glucose can be transformed into other value-added chemicals such as hydroxymethylfurfural [139]. Additionally, during acid-catalysed pretreatment, only low amounts of lignin are removed in the form of soluble fragments. A lot of research is currently underway to obtain other potential chemicals using acid hydrolysis [140]. On the other hand, hydrolysis with diluted acid required a high range of temperature to achieve the optimum yield of sugar and degradation of cellulose [138]. Even though feedstock hydrolysis with concentrated and diluted acid is efficient, while it is regarded as a suitable technique for sugar production, it results in sugar degradation product, which severely limits the cell growth and sugar yield.

Since acid hydrolysis has many drawbacks, enzymatic hydrolysis is the most convenient solution because it is a gentle and environmentally friendly method that uses less energy [137]. According to Heng et al., which achieved 87% yield of total reducing sugars using KOH, the general purpose of utilising either acid or alkali hydrolysis enhanced the accessibility of cellulose for enzymatic hydrolysis [89]. Generally, the maximum yield of sugar obtained from enzymatic hydrolysis is greater compared to acid hydrolysis [89]. However, lignin may cause an inhibitory effect depending on the type of pretreatment used [141].

Endoglucanase, exocellobiohydrolase, and β-glucosidase work together to hydrolyse the cellulosic component in cellulosic hydrolysis [142]. According to Maitan-Alfenas et al., endoglucanase cleaves cellulose’s β-1, 4 connections at exposed sites to produce new reducing ends, while the exocellobiohydrolase hydrolyses units of cellobiosyl from ends of non-reducing, specifically. Finally, cellobiose is converted to glucose by β-glucosidase [137].

Unlike cellulose, hemicellulose hydrolysis is more difficult because it involves the use of many different enzymes with different specificity as well as modes of action to achieve monosaccharides from hydrolysis. Exo-enzymes cut oligosaccharide and disaccharide endings, while endo-enzymes cut the bonds whereas the remaining enzymes hydrolyse glucuronoyl and acetyl residues [143].

### 5.3. Bacterial Fermentaion of PHA 

PHA is produced as inclusion bodies inside cells, and their development is primarily influenced by cell densities [144]. Several studies have been reported effective fermentation strategies in order to increase the PHA yields using batch, fed-batch, and continuous fermentation [145]. However, many factors influence the approach, including the carbon source, type of bioreactor, and form of culture.

Due to its low cost and versatility, batch fermentation is an effective and commonly applied method for biopolymer manufacturing. It is defined as a closed system in which the substrate and other components are added at the start of the experiment. The substrate and other components react inside the reactor, and the product can be extracted once the reaction is complete [146]. However, batch fermentation has lower productivity than other fermentations because of the deterioration of accumulated PHA after the carbon source has been fully used, resulting in a decrease in PHA material. When a high concentration of substrate is added to a batch culture to resolve carbon exhaustion, the growth and output yield are inhibited [145]. Also, according to Amache, the intermediate analysis cannot be performed since no sample can be taken out during the reaction period [145].

Fed-batch fermentation is the favoured fermentation in the industry, as well as the most efficient way to achieve high cell density cultivation, high amount of yield, as well as performance. As a result, it is commonly used in microbial fermentation to produce PHA [147]. In fed-batch fermentation, cells are grown in a batch mode until the end of the exponential stage is achieved, then placed in a bioreactor with a shortage of essential and carbon sources [148]. 

Fed-batch fermentations prevent the problem of bacteria starvation at the end of the reaction, which is common in batch reactors. Fed-batch reactors also make it possible to add more substrates to the system during the cultivation process. This causes the substrate to be at the proper concentration for fermentation. pH, substrate concentration, and dissolved oxygen are some of the other parameters that fed-batch fermentation reactors can regulate. Controlling these variables allows for high cell density and PHB output in the end [146].

Many studies have been conducted using hydrolysates of lignocellulosic biomass to produce PHA by *Pseudomonas resinovorans*, *Bacillus megaterium*, *Burkholderia cepacia*, and *Burkholderia sacchari* using fed-batch fermentation [109,149]. For instance, Cesário et al. used sources from wheat straw hydrolysate for PHB production and achieved very high PHA accumulation and biomass (135.8 g/L and 105.0 g/L) using this fermentation technique [99].

Chemostat cultivation, also known as continuous fermentation, is a common operation technique for the production of PHA in which the culture medium, substrate, and other requirements are continuously pumped into the bioreactor [143]. The substrate is continuously fed in abundance, while one or more nutrients are held in famine. It has a high degree of control and is based on a specified growth rate that may be changed using the dilution rate. PHA build-up, yield, and productivity will all benefit from the continuous cultivation technique but with a greater chance of contamination [145].

## 6. PHA in Commercial Scale

PHA has received a lot of attention in recent years. They are undeniably valuable materials with significant properties [150]. The main disadvantage of PHA is that they are more costly than petroleum-based plastics. For instance, the microbial and commercial PHB produced is still around three times the cost of petroleum-based plastics. PHB cost around USD 3.50 per kg in 2018, while petroleum-based plastics cost around USD 1.20–1.30 per kg as reported in the same study [151]. There have been reports on PHA production on a commercial scale, as well as commercial PHA manufacturers and their capacity [62,152,153]. The cost of feedstocks used as a source of carbon for microbial development determines the overall costs of PHA in large. Another major barrier to commercial PHA production is maintaining the conditions of bacterial growth are optimal as well as maximising PHA titre, cell accumulation, and productivity.

Despite several efforts to reduce the major barriers to production by using renewable low-cost substrates and continuously developing and assessing the biopolymer’s sustainable production, only a few companies are pushing forward to produce PHA on a commercial scale in the longer term (Table 8).

PHA production was projected to be 54 kilotonnes in 2014, and it is predicted to face a five-fold increment by 2020 [113]. Due to the demand from the market and healthcare industries for renewable resources, as well as recent improvements in PHA manufacturing technologies in 2017, the PHA market is expected to expand at a compound annual growth rate of about 6.3% per 10 years, with almost USD 119.15 million by 2025 (Table 9). The most popular, widely developed, and best-characterised homopolymer is PHB [157].

## 7. Challenges, Opportunities, and Way Forward

Bacteria that have been genetically modified can collect more PHA in their cellular biomass and produce single selected monomers rather than a mix of copolymers. The cost of downstream processes associated with PHA recovery and extraction should also be reduced by employing more cost-effective, modern procedures. The completed application of the finished products determines the efficiency of the operations [160]. Bioplastics are gaining popularity in the biomedical field, and for that reason, the pure form of a product is desired, which can be obtained through a cost-effective downstream process. The use of modern biological methods, such as synthetic biology and genetic modification, boosts the production of non-toxic PHA bacteria. These non-toxic PHA could be used in a variety of biomedical applications with ease. On the other hand, toxin-free PHA derived from bacteria, require further steps. The cost of PHA manufacturing is increased even more by the post-cleaning procedures. Researchers should concentrate their efforts on obtaining high amounts of yield, high-purity and toxin-free PHA from bacteria that are often used these days in commercial-scale PHA synthesis. For enhanced accumulation of PHA polymers, the selection of using genetically altered Gram-positive bacteria should be investigated. Gram-positive bacterial strains can be used in the cost-effective medical-grade PHA production from a laboratory to a commercial scale since PHA from Gram-positive bacteria have been reported to have low levels of immunogenic lipopolysaccharide (LPS) [161].

Additionally, lower PHA production costs can enable a new platform for their applications in biological disciplines, as well as modify the community’s perspective and standards. PHA can also revolutionise established pharmacological procedures, and protein immobilisation on biopolymers could broaden its emerged applications. This innovative, biodegradable, and biocompatible polymer is a promising future option, with the potential to partially replace synthetic petroleum-based plastics even in cosmetics [162].

Instead of a single kind of PHA, microbial production creates a mixed monomeric composition, but separating pure monomers from complicated mixtures is a major difficulty. To make the biopolymers commercially sustainable, researchers are working to reduce the cost of production of PHA from synthesis to procedure in the aftermarket. For the cost-effective manufacture of PHA, a variety of procedures and strategies have been examined, including various carbon sources and energy, variable yield product, duration, the procedure of extraction, and purity of the product [21]. For instance, a product made from PHA for biomedical use would need excellent purity. If the product is made as a one-time-use disposable, the cost of downstream processing will be the most important consideration. Moreover, as mentioned previously, bioplastic products produced from the fermentation of renewable carbon materials can degrade or be composted within a month on average, depending on their applications, into carbon dioxide and water. The waste PHA products are subsequently either composted, recycled, or converted into PHA carbon feedstocks, allowing the practice of the cradle-to-cradle concept feasible [7,8,15]. However, durable products still require more durable materials (e.g., metal, glass) than PHA albeit short-life or single-use products can be substituted by PHA. Additionally, although PHA can help to solve the reduced use and manufacture of fossil-based plastics, another limitation of material substitution by PHA is the downstream problem of demand for durable yet environmentally friendly materials.

It is beneficial to use lignocellulosic materials in the development of new products in order to better address environmental concerns [163]. In this scenario, the biorefinery concept is gaining favour to turn waste into profit with minimal environmental impact [164]. The procedure is cost-effective since environmental waste is used as a medium for microorganism development and the synthesis of innovative, valuable biopolymers [37]. Commercially desirable is a life cycle study of microbial PHA production utilising LF as the substrate [165]. A variety of LF that acts as carbon sources create a wide range of polymer yields and beneficial microorganisms.

## 8. Conclusions and Recommendation

PHA has received a lot of attention in the biodegradable polymer market because it could be a long-term solution for damaging fossil-based polymers. However, because carbon sources account for half of the production cost, price is seen as the most significant barrier to commercialization. As a result, it is critical to look at low-cost, renewable, long-term, and alternative feedstocks to help bioplastic compete with its equivalent. As a result, LF has a few advantages over ordinary sugar and starch-based crude biomass, and it is expected to be one of the most important biorefinery bio-resources.

The conventional processes used for LF to use as a carbon source for PHA-producing bacteria are clearly discussed in this review. However, the generation of PHA from lignocellulose is still in its infancy, but the future seems bright. Improved fermentation processes using low-cost forestry and agricultural waste could completely transform the global biopolymer industry, allowing production costs to compete with petroleum-based polymers while also providing beneficial biodegradable and biocompatible properties, environmental sustainability, and new biopolymer markets with novel applications. LF looks to have promising potential for long-term PHA production as an alternative to conventional feedstocks. To investigate this theory, more research on other sugar sources is required.

Various bacterial strains can utilise the LF to produce PHA. The ability of microorganisms to consume both the hexoses and pentoses released from lignocellulosic biomass and convert these sugars into products at high conversion yields is critical to the economic feasibility of using lignocellulosic hydrolysates as carbon sources for biologically producing biopolyesters. As a result, powerful bacteria capable of fermenting various forms of sugar are required for greater PHA production. This could make it easier for these important, environmentally friendly polymers to compete with petroleum-based plastics and, as a result, partially replace them in some applications.

Based on the work presented in this review, more improvements in the fields of downstream processing and substrate pre-treatment are needed in the future to see the biopolymer sector in a broader light.

## Figures and Tables

**Table 1:** The global increase in plastic production from 1960 to 2020 (Data sources from Ref. [21]).

Year	Global Plastic Production (Mt)
1960	1.5
2000	100
2010	250
2018	330
2020	400

**Table 2:** Classification of polyhydroxyalkanoate (PHA) based on the number of carbons.

Group	Number of Carbon (C) Atoms	Examples	References
Short chain length (SCL)	3–5	Poly(3-hydroxybutyrate)	[37]
		Poly(4-hydroxybutyrate)	[40]
		Poly(3-hydroxyvalerate)	[41]
		Poly(3-hydroxybutrate-*co*-3-hydroxyvalerate)	[41]
Medium chain length (MCL)	6–14	Poly(3-hydroxyhexanoate)	[42]
		Poly(3-hydroxyoctanoate)	[41]
		Poly(3-hydroxyhexanoate-*co*-3-hydroxyoctanoate)	[43]
Long chain length (LCL)	Above 14	Poly(3-hydroxypentadecanoate)	[31]
		Poly(3-hydroxyhexadecanoate)	[44]

**Table 3:** Production of polyhydroxyalkanoate (PHA) using various strains of bacteria and substrates.

Strain	Substrates	PHA Type	References
*B. subtilis* RS1	Pretreated molasses	PHA	[51]
*H. mediterranei*	Enzyme hydrolysed cheese whey	P(3HBHV)	[52]
*H. halophilia*	Diluted acid pretreated spend coffee ground	P(3HB)	[53]
*C. necator* H16	Waste frying rapeseed oil	P(3HB)	[54]
*C. necator* DSM 428	Oil from spend coffee ground	SCL PHA	[55]
*C. necator* DSM 7237	Crude glycerol, sunflower meal hydrolysates and levulinic acid	P(3HBHV)	[56]
*P. aeruginosa* STN-10	Frying oil	PHA	[57]
*H. mediterranei*	Olive oil wastewater with inhibitory polyphenols	PHA	[58]
*Halomonas* i4786	Fruit processing wastewater	PHA	[59]
Fresh activated sludge	Organic fraction of municipal solid waste (MSW)	PHA	[60]
*B. sacchari* DSM 17165	Wheat straw hydrolysate	P(3HB)	[61]
*P. sacchari* IPT 101	Softwood hemicelulose hydrolysate	P(3HB)	[62]
*Burkholderia* sp. F24	Xylose and levulinic acid	P(3HBHV)	[63]
*C. basilensis* CGMC 4240	Kraft lignin	P(3HB)	[64]
*P. putida* KT2440	Alkaline pretreated liquor (APL)	MCL PHA	[65]
*C. necator* DSM 545	APL	P(3HB)	[65]

P(3HBHV), poly(3-hydroxybutyrate-*co*-hydroxyvalerate).

**Table 4:** Types of lignocellulosic feedstocks (LF) commonly used for bioconversion (adapted and modified from Hadar [87]).

Category	Description	Examples
Woody biomass	Divided into hardwoods and softwoods Softwoods originate from gymnosperms and conifers Hardwoods originate from angiosperms	Wood chips Stumps and dead tree materials Sawdust
Perennial bioenergy crops	Perennial crops have a lifespan of more than two years Grown specifically for use as a biomass resource	*Miscanthus* sp. Switchgrass Sorghum
Agricultural residues	Parts of the crop that cannot be used for the food or food derivatives Often left on-site during harvesting, or collected as an output from mills	Straw (wheat, barley, rice) Husks (barley, rice) Corn stover Empty fruit bunch (oil palm) Sugarcane bagasse
Municipal solid waste (MSW)	Biodegradable organic components from household waste Not as ideal as other types of biomasses but useful in regions where crops are scarce	Paper and cardboard waste Solid kitchen and garden waste

**Table 5:** Summary of polyhydroxyalkanoate (PHA) production from lignocellulosic feedstocks (LF).

LF Material	Microorganisms	Type of PHA	PHA Concentration (g/L)	PHA Performance (%)	References
Grass biomass	*Pseudomonas* strains	MCL PHA	0.3	33	[104]
Coir pitch	*Azotobacter beijerinickii*	PHB	2.4	48	[105]
Wheat bran hydrolysate	*Bacillus sacchari*	PHB	105.0	72	[99]
Spent coffee ground	*Burkholderia cepacia*	P(3HB-*co*-3HV)	2.69	54.79	[106]
Corn stover	*Paracoccus* sp. LL1	PHB	9.71	72	[107]
Rice husk hydrolysate	*Bacillus mycoides*	P(3HB-*co*-3HV)	0.39	21.6	[108]
Wood hydrolysate	*Burkholderia cepacian*	PHB	8.72	51.4	[91]
Fruit pomace and waste frying oil	*Pseudomonas resinovorans*	MCL PHA	1.8	12.4	[109]
Jackfruit seed powder	*Bacillus thuringiensis* IAM 12077	PHB	4.03	51.3	[110]
Mango peel	*Bacillus thuriengiensis* IAM 12077	PHB	8.03	51.7	[110]
Water hyacinth	*Cupriavidus necator*	PHB	7	58	[111]
Sunflower hydrolysate	Recombinant *R. eutropha*	PHB	7.86	72.53	[112]
Wheat bran	*Ralstonia eutropha* NCIMB 11599	PHB	14.82	62	[96]
Waste office paper	*Ralstonia eutropha* NCIMB 11599	PHB	4.45	57.52	[113]
Lignin	*C. necator* DSM 545	PHB	4.5	-	[83]
Pinus radiata wood	*Novosphingobium nitrogenifigens* and *Sphingobium scionense*	PHB	0.39	32	[114]
Sugar cane bagasse, wheat bran and rice bran hydrolysate, with unhydrolyzed corn starch	*Bacillus* sp. CFR-67	PHBV	5.9	-	[115]
Rice straw hydrolysate	*Bacillus firmus* NII 0830	PHA	1.7	-	[116]
Sugar maple hemicellulosic hydrolysate	*Burkholderia cepacian* ATCC 17759	PHA	8.7	51	[91]
Rice straw	*Ralstonia eutropha*	PHB	11.42	-	[100]
Lignin	*Ralstonia eutropha* H16	PHA	0.6	-	[83]
Wastewater hydrolysate	*Burkholderia sacchari*	PHB	-	44.2	[80]
Cane bagasse	*Bacillus* sp.	PHB	5.00	55.6	[24]
Rice husk	*Burkhaderia cepacian* USM (JCM 15050)	PHB	4.85	40.0	[24]
Corn cob	*Bacillus* sp.	PHB	4.80	51.6	[24]
Teff straw	*Bacillus* sp.	PHB	3.20	38.6	[117]
Ragi bran	*Bacillus thuringiensis* IAM 12077	PHB	0.32	26.7	[117]
Rice bran	*Bacillus thuringiensis* IAM 12077	PHB	0.21	31.8	[117]
Wood hydrolysate	*Paraburkholderia sacchari*	PHB	34.5	58	[118]
Tequila agave bagasse hydrolysate	*Burkholderia sacchari*	PHB	24	-	[119]
Spruce sawdust hydrolysate	*Burkholderia cepacia*	PHB	1.45	74.7	[120]
Spent coffee grounds hydrolysate	*Bacillus megaterium*	PHB	1.7	51	[121]

P(3HB-*co*-3HV), poly(3-hydroxybutyrate-*co*-3-valerate); PHBV, polyhydroxybutyrate-*co*-valerate.

**Table 6:** The various types of pretreatment methods involved in the conversion of lignocellulosic feedstocks (LF) to polyhydroxyalkanoate (PHA).

Physical	Physico-Chemical	Mechanico-Chemical	Thermo-Chemical	Chemical	Biological
Miling Comminution Ultrasound Microwave Irradiation	Liquid hot water	Steam explosion (SE) Ammonia fibre expansion (AFEX)	Organosolv Acid and alkaline hydrolysis	Oxidation delignification by peroxide Ozonolysis Wet oxidation Ionic liquid (IL)	Microbial enzyme (delignification)

**Table 7:** Advantages and disadvantages of various types of feedstocks pretreatments.

Pretreatment	Advantages	Disadvantages	Reference
**Physical**			
Comminution	Decreases crystallinity of cellulose Improves mass transfer of bulk material	Requires high energy input	[133]
Irradiation	Disrupts lignin Decreases crystallinity of cellulose	Hazardous (high radiation doses)	[134]
Ultrasound	Moderate treatment conditions	Superficial effect on biomass	[135]
Microwave	Low power consumption and low-cost treatment Effective when applied in combination with chemicals Treatment times are proportional to the power of the microwave oven Efficient and environmentally friendly Less or no solvents are required	Energy-intensive and expensive	[136]
**Chemical**			
Ozonolysis	Disrupts lignin and hemicellulose Can be performed at lower temperatures Reduces formation of inhibitory compounds	Requires large amounts of ozone High cost of material and equipment	[80]
Ionic liquid (IL)	Have low vapour pressures, good thermal stability, and various combination of ion Dissolves cellulose and lignin	-	[80]
Wet oxidation	Organic molecules are decomposed into CO_2_, H_2_O, and simpler and more oxidised organic compounds The residual solid is cellulose with low lignin content	Generation of low amounts of furfural and hydroxymethylfurfural (inhibitors in the fermentation) Large amounts of hemicellulosic sugars are lost Exothermal process, requiring control of process parameters	[80]
**Physico-chemical**			
Superheated steam	Does not require catalysts Environmentally friendly	Low sugar yields from hydrolysis when used alone	[136]
Liquid hot water	No addition of chemicals for neutralization Produce lower amount of inhibitory products Dissolve cellulose Removes hemicellulose and lignin	-	[80]
**Mechanico-chemical**			
Steam explosion (SE)	Solubilises hemicellulose Initiates an autocatalytic reaction Improves accessibility to cellulose	Generates inhibitory compounds	[136]
Ammonia fibre expansion (AFEX)	Solubilises hemicellulose Reduces loss of cellulose Allows recovery of chemical	High cost of equipment Not efficient on biomass with high lignin content	[136]
**Thermo-chemical**			
Acid	Solubilises hemicellulose Disrupts lignin Increases accessibility of cellulose	Highly corrosive and damaging to equipment Generates inhibitory compounds	[136]
Alkaline	Removes lignin Reduces formation of inhibitory compounds Can be performed at lower temperatures	Formation and deposition of salts on the substrate Long residence time for lower temperatures	[112]
Organosolv	Solubilises hemicellulose and lignin Initiates an autocatalytic reaction Allows recovery of chemicals	Requires thorough washing of pretreated material	[80]
**Biological**			
Microbial enzymes (delignification)	Enhances enzymatic hydrolysis Degrades lignin and cellulose Low-capital cost and energy input High yield without generating inhibitory products	Very low hydrolysis rate Long pretreatment time and degradation	[136]

**Table 8:** Polyhydroxyalkanoate (PHA) production at the global level [154,155,156].

Company	Country	Year of Starting	Product	Substrate	Production Volume (Tonne/Year)
Kaneka	Japan	1949	PHBH	Plant	50,000
Novamont	Italy	1989	PHA	Natural waste	-
Metabolix	Massachusetts	1992	PHA, PHB	Switchgrass	50,000
Rodenburg Biopolymer	The Netherlands	2000	PHA	Renewable materials	-
TianAn Biological Material Co. Ltd.	China	2000	PHBV	-	10,000
Danimer Scientific	Georgia	2007	MCL PHA	-	272,000
Bio-On	Italy	2007	PHA	Renewable waste	10,000
Newlight Technologies, LLC	US	2007	PHA	Greenhouse gases	-
Vinmar	-	2008	PHA	Greenhouse gases	-
NAFIGATE Corporation	Czech	2015	P(3HB)	Coconut peeling milk	-

PHBH, polyhydroxybutyrate-*co*-hydroxyhexanoate.

**Table 9:** Global production of polyhydroxyalkanoate (PHA) from 2015 to 2025 (Data sources from Refs. [80,158,159]).

Year	Global PHA Production (Million USD)
2015	70
2016	75
2021	81.8
2025	119.15

## Data Availability

Not applicable.
